# Updated research gaps on ending child marriage and supporting married girls for 2020–2030

**DOI:** 10.1186/s12978-021-01176-x

**Published:** 2021-07-20

**Authors:** Marina Plesons, Ellen Travers, Anju Malhotra, Arwyn Finnie, Nankali Maksud, Satvika Chalasani, Venkatraman Chandra-Mouli

**Affiliations:** 1 UNDP-UNFPA-UNICEF-WHO-World Bank Special Programme of Research, Development and Research Training in Human Reproduction (HRP) and the World Health Organization, Geneva, Switzerland; 2 Independent Consultant, Dublin, Ireland; 3 United Nations University, International Institute for Global Health, Kuala Lumpur, Malaysia; 4 Girls Not Brides: The Global Partnership To End Child Marriage, London, England; 5 UNICEF, New York, USA; 6 UNFPA, New York, USA

## Abstract

Over the past 25 years, tremendous progress has been made in increasing the evidence on child marriage and putting it to good use to reduce the prevalence of child marriage and provide support to married girls. However, there is still much to be done to achieve the Sustainable Development Goal target 5.3 of ending child marriage by 2030, and to meet the needs of the 12 million girls who are still married before age 18 each year. To guide and stimulate future efforts, the UNDP-UNFPA-UNICEF-WHO-World Bank Special Programme of Research, Development and Research Training in Human Reproduction, the World Health Organization, the UNICEF-UNFPA Global Programme to End Child Marriage, and *Girls Not Brides: The Global Partnership to End Child Marriage* convened an expert group meeting in 2019 to: (1) review the progress made in building the evidence base on child marriage since the publication of research priorities in this area in 2015, (2) identify an updated set of research priorities for the next ten years, and (3) discuss how best to support research coordination, translation, and uptake.

This article provides a summary of the progress made in this area since 2015 and lists an updated set of research gaps and their rationale in four key areas: (1) prevalence, trends, determinants, and correlates of child marriage; (2) consequences of child marriage; (3) intervention effectiveness studies to prevent child marriage and support married girls; and (4) implementation research studies to prevent child marriage and support married girls. It also highlights a number of calls-to-action around research coordination and knowledge translation to support the emerging and evolving needs of the field.

## Résumé

Au cours des 25 dernières années, d'énormes progrès ont été réalisés pour mettre à jour les données concernant le mariage des enfants et les mettre à profit pour réduire la prévalence du mariage des enfants et apporter un soutien aux filles mariées. Cependant, il reste encore beaucoup à faire pour atteindre la cible 5.3 de l'Objectif de Développement Durable consistant à mettre fin au mariage des enfants d'ici 2030 et pour répondre aux besoins des 12 millions de filles qui sont encore mariées avant l'âge de 18 ans chaque année. Guider et stimuler les efforts futurs, tel est le Programme Spécial de Recherche de la Banque Mondiale PNUD-FNUAP-UNICEF-OMS, du Développement et la Formation à la Recherche en Reproduction Humaine, de l'Organisation Mondiale de la Santé, du Programme Mondial UNICEF-FNUAP pour mettre fin au mariage des enfants et «  Des filles pas des Mariées  » : le Partenariat Mondial pour mettre fin au mariage des enfants a convoqué une réunion de groupe d'experts en 2019 pour : 1. passer en revue les progrès accomplis dans la constitution de la base de données factuelles sur le mariage des enfants depuis la publication des priorités de la recherche dans ce domaine en 2015, 2. identifier un ensemble actualisé de priorités de recherche pour les dix prochaines années, et 3. discuter de la meilleure façon de soutenir la coordination, la traduction et la compréhension de la recherche.

Cet article résume les progrès réalisés dans ce domaine depuis 2015 et répertorie sous forme de mise à jour un ensemble de lacunes dans la recherche et leur justification dans quatre domaines clés: 1) prévalence, tendances, déterminants et corrélatifs du mariage des enfants; 2) conséquences du mariage des enfants; 3) études ayant trait à l'efficacité des interventions pour prévenir le mariage des enfants et soutenir les filles mariées; et 4) études de recherche sur la mise en œuvre pour prévenir le mariage des enfants et soutenir les filles mariées. Il met également en évidence un certain nombre d'appels à l'action autour de la coordination de la recherche et de l'application des connaissances pour répondre aux besoins émergents et à l’évolution de ce domaine.

## Resumen

En los últimos 25 años se ha avanzado enormemente y existe ahora más evidencia sobre el matrimonio infantil, y sobre como se debe poner la evidencia en práctica para reducir la prevalencia del matrimonio infantil y proporcionar apoyo a las niñas casadas. Sin embargo, aún queda mucho por hacer para alcanzar la meta 5.3 de los Objetivos de Desarrollo Sostenible de poner fin al matrimonio infantil para 2030, y para satisfacer las necesidades de los 12 millones de niñas que cada año se casan antes de los 18 años. Para orientar y estimular los esfuerzos futuros, el Programa Especial de Investigación, Desarrollo y Formación de Investigadores en Reproducción Humana del PNUD-FNUAP-UNICEF-OMS-Banco Mundial, la Organización Mundial de la Salud, el Programa Mundial para la Eliminación del Matrimonio Infantil de UNICEF-FNUAP y Girls Not Brides: La Alianza Mundial para Acabar con el Matrimonio Infantil convocó una reunión de un grupo de expertos en 2019 para: 1. Revisar los progresos realizados en la construcción de la base de evidencias sobre el matrimonio infantil desde la publicación de las prioridades de investigación en este ámbito en 2015, 2. Identificar un conjunto actualizado de prioridades de investigación para los próximos diez años, y 3. Debatir la mejor manera de apoyar la coordinación, la traducción y la aceptación de la investigación.

Este artículo ofrece un resumen de los progresos realizados en este ámbito desde 2015 y enumera un conjunto actualizado de lagunas de investigación y su justificación en cuatro áreas clave: 1) prevalencia, tendencias, determinantes y correlaciones del matrimonio infantil; 2) consecuencias del matrimonio infantil; 3) estudios de eficacia de las intervenciones para prevenir el matrimonio infantil y apoyar a las niñas casadas; y 4) estudios de implementación para prevenir el matrimonio infantil y apoyar a las niñas casadas. También destaca una serie de llamados a la acción en torno a la coordinación de la investigación y la traducción de conocimientos para apoyar las necesidades emergentes y en evolución del campo.

Over the past 25 years, considerable progress has been made towards the goal of ending child marriage [1, 2]. Laws banning child marriage and national strategies to prevent it are in place in a growing number of countries [1]. Alongside a rapid increase in efforts by civil society organizations, a UN-coordinated, multi-donor, global programme is operational and there are government-led/supported efforts in a growing number of countries [1]. Within such programming, there is greater attention to understanding and addressing the structural drivers of child marriage [1]. These efforts, alongside broader improvements in overall economic situations, access to educational and employment opportunities, and urbanization, have contributed to declines in the prevalence of child marriage [1–3]. However, there is still much to be done to achieve the Sustainable Development Goal target 5.3 of ending child marriage by 2030, and to support the 12 million girls who are still married before age 18 each year [4].

In 2015, the UNDP-UNFPA-UNICEF-WHO-World Bank Special Programme of Research, Development and Research Training in Human Reproduction (HRP), the World Health Organization (WHO), UNICEF, and *Girls Not Brides: The Global Partnership to End Child Marriage* published research priorities on ending child marriage and supporting married girls [5]. Guided by these research priorities, evidence on child marriage has expanded substantially, highlighting the scale, impact, and nature of child marriage [2, 6]. This evidence has, in turn, contributed to the progress described above [1]. However, a number of evidence gaps remain and new areas of research interest have emerged. Likewise, there is a need to strengthen the bridge between the producers and consumers of research on child marriage to accelerate evidence-informed policy-making and programme implementation. In recognition of these needs, HRP, WHO, the UNICEF-UNFPA Global Programme to End Child Marriage, and *Girls Not Brides* convened an expert group meeting in 2019 to:

1. review the progress made in addressing the research priorities published in 2015 [5],
2. identify an updated set of research priorities for the next ten years [7], and
3. discuss how best to support research coordination, translation, and uptake [7].

To achieve objectives 1 and 2, the meeting drew on a systematic review and a comprehensive scoping review, both covering 20 years of the evidence base, and the experiences and viewpoints of the technical experts in attendance [2, 6, 8]. To achieve objective 3, it took advantage of the unique opportunity that the meeting presented by bringing together consumers of research with its producers to discuss what could be done better to improve research coordination, translation, and uptake.

This article provides a summary of the progress made in this area since 2015, lists an updated set of research gaps in four key areas and the rationale for addressing them, and highlights a number of calls-to-action around research coordination and knowledge translation to support the emerging and evolving needs of the field.

## Progress since 2015



The evidence base on child marriage has grown substantially in the past 5 years [2, 6, 8]. Specifically, we have more clarity on its prevalence, trends, determinants, and correlates [6]. Child marriage is most common in South Asia and sub-Saharan Africa, with significant declines in some countries and stagnation or even increases in others. Across most countries, intersecting vulnerabilities put girls living in poor and rural communities at greatest risk of child marriage and its consequences, while also being least served by prevention and mitigation efforts.

With regard to determinants, while there are contextual and temporal differences, there are a number of important similarities across regions, including: economic motivations, insecurity, unequal gender-power structures including those that relate to control of girls’ sexuality, and lack of opportunities and life options for girls. In combination, these drivers often manifest as gendered social norms. Girls’ education—especially at the secondary level—is the most consistent protective factor against child marriage. Urban residence, being part of educated and financially secure families, and having better employment prospects are also protective factors in most settings. In some contexts, other household dynamics, such as female-headed households or the presence of older sisters, also act as protective factors.

With regard to consequences, most evidence focuses on maternal and perinatal health consequences of child marriage, followed by the social, health, developmental and intergenerational impacts. Less studied are broader impacts on health and social well-being, economic costs, and consequences for younger adolescent girls.

Finally, with regard to interventions to prevent child marriage, there is increasing evidence supporting the use of interventions that enhance girls’ human capital and opportunities (i.e., providing conditional cash transfers for girls’ schooling, expanding labor market opportunities, and fostering the development of livelihood skills) [2]. Meanwhile, conditional cash transfers for delaying marriage, and gender rights and life skills training have demonstrated mixed results, and unconditional cash transfers appear to be ineffective at preventing child marriage [2].

## Research gaps and their rationale

There was general consensus from the scoping and systematic reviews and the discussions during the expert group meeting that while the expansion of research has been beneficial in increasing investment and improving action on child marriage, there are still a number of gaps in our understanding of child marriage, especially in areas that have either been neglected or emerged in sharper focus over recent years. For example, despite more robustly documented rates of child marriage, we have less clarity on rates and trends at sub-national levels, which would allow for more precise targeting of programmes. Similarly, beyond the broader role of macro-level shifts, we have limited understanding of the factors that have led to large scale declines. Likewise, we lack evidence on the effectiveness, cost, and cost-effectiveness of a critical package of interventions to prevent child marriage and support married girls, and what it takes to deliver these interventions at scale and in a sustainable and equitable manner. With these priorities in mind, the meeting participants outlined the following gaps for the research agenda over the next 10 years.

### Prevalence, trends, determinants, and correlates of child marriage

1. Carry out sub-national and sub-population level data collection and analyses of prevalence, trends, determinants, and correlates of child marriage, and ensure that such analyses are disaggregated by both sex and age and include year-by-year analysis, where possible.
  *Rationale: To make the case and to focus investments more precisely, to understand where within countries and in which sub-populations declines are happening (or not happening) and why.*
2. Carry out such analyses during and in the aftermath of humanitarian crises.
  *Rationale: To better understand short- and long-term implications of humanitarian crises on child marriage and potential responses.*
3. Map shifts in prevalence of child marriage against changing macro-, meso-, and micro-level social and economic trends.
  *Rationale: To understand which larger and cumulative societal shifts contribute to changes in child marriage over time and how these might by leveraged or accelerated for reductions at scale.*
4. Assess timing and prevalence of pregnancy and childbearing in relation to child marriage.
  *Rationale: To better anticipate concurrence and lags in how these correlated outcomes change and to inform the design of approaches to address them.*
5. Define measures and approaches to assess the attributes and quality of marriages, with a particular focus on shifts in power and girls’ empowerment.
  *Rationale: To understand trends in the attributes and quality of marriages and the status and satisfaction of girls/young women in their marriages.*

### Consequences of child marriage

1. Carry out analyses (where they do not already exist) of short- and long-term consequences of child marriage for girls across their life course (e.g., related to childbearing, health including mental health, education, economic well-being/opportunities, family size and structure, power relations, violence, and social support systems), for the men and boys whom they marry, and for their children and families.
  *Rationale: To inform more effective advocacy, prevention, and support efforts, and to forge links with programmes addressing the needs of girls and women more broadly.*
2. Carry out analyses of marriage transitions following child marriage/early unions (e.g., household formation or dissolution, separation, divorce, widowhood, and desertion).
  *Rationale: To better inform efforts to support the needs of girls whose child marriage/union unravels.*
3. Carry out analyses of short- and long-term consequences of child marriage for girls and for their families in humanitarian crises or refugee contexts (including with temporary/contractual arrangements).
  *Rationale: To better inform advocacy, prevention and support efforts in humanitarian crises or refugee contexts.*

### Intervention effectiveness studies to prevent child marriage and support married girls

1. Carry out intervention effectiveness studies/evaluations to assess the impact of specific interventions/approaches to delay marriage and support married girls, targeted at different groups (e.g., girls, boys/men, parents, and communities) and in specific contexts.
  *Rationale: To fill gaps in evidence in relation to specific interventions/approaches where the existing evidence is mixed, and in contexts where few such studies have been carried out.*
2. Carry out a broader set of intervention effectiveness studies/evaluations to assess the comparative impact and implementation of single interventions versus comprehensive packages of interventions.
  *Rationale: To determine the relative cost, implementability, effectiveness, and scalability of single interventions compared to multi-component packages of interventions.*
3. Carry out intervention effectiveness studies/evaluations to assess the impact of interventions aimed at bringing about change at the individual or community levels versus those aimed at doing so at the systems level.
  *Rationale: To determine whether interventions aimed at bringing about change at the system level should be applied in child marriage prevention and mitigation.*

### Implementation research studies to prevent child marriage and support married girls

Delivery platforms and approaches:

1. Map functional and at-scale platforms that could be leveraged to intensify/accelerate the delivery of interventions (1) to prevent child marriage and (2) to support married girls and their children in humanitarian crisis and non-crisis settings.
  *Rationale: To make the best use of available platforms to extend the reach of effective interventions.*
2. Identify approaches for closer integration of child marriage activities within delivery platforms in other sectors (e.g., education and employment) and health issues (e.g., FGM and VAW).
  *Rationale: To ensure greater effectiveness and efficiency of activities through meaningful collaboration with key sectors.*
3. Identify approaches to address the needs and problems of the hardest-to-reach girls and communities.
  *Rationale: To assess and learn how programmes can practically respond to the challenge of reaching those in the most remote rural areas and in the lowest wealth quintiles.*
4. Assess the cost, scalability, and sustainability of single interventions versus multicomponent packages of interventions.
  *Rationale: To learn more granularly the challenges and successes of undertaking these approaches, and to determine which are practical, feasible, and effective at scale.*

Role of civil society organizations in scale-up efforts:

1. Assess what capacities civil society organizations will need to support the scale-up of child marriage activities with the government and/or private sector, both in contexts where the government capacity is weak, and in those in which it is not, but where the government is not playing a leading role.
2. Assess the sustainability and impact of civil society organizations in scale-up efforts.
  *Rationale (for items 1 and 2): While civil society organizations have and continue to play the role of advocate and innovator, they are increasingly being called upon to contribute to scale-up efforts.*

Investment, policies, and large-scale programmes:

1. Assess investment needs and gaps for preventing child marriage and supporting married girls.
  *Rationale: To determine the required funding for preventing child marriage and supporting married girls, against the available funding.*
2. Document and evaluate the scale and time-frame of scaled-up programmes, what they did and how they did it, how much they cost, and their strengths and weaknesses.
  *Rationale: To draw out lessons through learning-by-doing with large-scale programmes in countries, which are seldom documented and evaluated.*
3. Assess whether a gender and equity lens is retained as initiatives move from small to large scale.
  *Rationale: To respond to the pressure that small projects face as they turn into larger programmes to reduce/limit the number of issues they address (or the way they address them) in the interest of scale.*
4. Assess the potential benefits and risks of interventions/approaches to create an enabling legal and social environment to address child marriage and respect girls’ rights, particularly their sexual and reproductive rights.
  *Rationale: To determine whether such interventions/approaches could lead to unintended negative effects (e.g., backlash from communities or driving the practice underground.)*
5. Assess the features of policy coalitions that have contributed to scale-up on issues with similar complexity to child marriage.
  *Rationale: To draw upon lessons learned in other areas of social change, health, and development.*
6. Assess what could be achieved through more coordinated action between United Nations agencies, funders, governments, and civil society organizations in a few specific countries.
  *Rationale: To determine the outcomes of stronger coordination and collaboration, and what it takes to bring this about.*

## Strengthening research coordination, translation, and uptake

Prioritizing, resourcing, and carrying out research:

1. At the global level, establish a research consortium to intentionally define and maintain a learning agenda on preventing child marriage and responding to the needs of married girls.
2. At the country level, bring together researchers, international agencies, government, and civil society organizations to support the establishment of similar mechanisms.
  *Rationale (for items 1 and 2): To ensure that research priorities are set collaboratively with the full involvement of stakeholders at global and country levels, and that they respond to the evolving needs of the field.*
3. Link funding for implementation of interventions to a research framework focusing on what factors support strategic interventions and investments.
  *Rationale: To ensure that opportunities for building the evidence base are used to their full potential.*
4. Build and enhance the capacity of researchers in low- and middle-income countries in designing and carrying out research, and in interpreting the ‘so-what’ of their research findings.
5. Invest in participatory action research to ensure the meaningful engagement of adolescents and communities in carrying out research.
  *Rationale (for items 4 and 5): To use the opportunity of existing research studies to build research capacity.*

Packaging and translating research findings:

1. Involve policy-makers and programme implementers in the design of research outputs.
  *Rationale: To ensure that the findings of research are presented in ways that respond to the needs of policy-makers and programme implementers.*
2. Systematically reach out to relevant sectors (e.g., health, education, gender, economic empowerment) to share research findings demonstrating the impact that investments in their sector could make to ending child marriage and mitigating its consequences.
  *Rationale: To encourage relevant sectors to make the contributions that they could to this area.*
3. Ensure that research findings are published in open access journals.
  *Rationale: To ensure accessibility of research findings.*
4. Develop briefs, blogs, infographics and slide sets for various audiences, as a complement to reports and journal publications.
  *Rationale: To ensure that non-research constituents have access to research findings in formats that are accessible and practical.*
5. Use available information exchange platforms to share and dissemination learnings on prevention and mitigation of child marriage.
6. Develop a ‘help-desk’ mechanism where policy and programme questions could be asked and responded to.
  *Rationale (for items 5 and 6): To expand the accessibility of research findings in a variety of formats and channels.*

## Conclusion

The participants of the 2019 expert group meeting called for a more purposive global learning agenda that responds to where the field is in different settings and contexts. They also called for efforts to bring together the consumers of research, those doing research, and those funding research on a regular basis. Finally, they stressed the importance of fully involving the countries and communities affected in setting research priorities and in carrying out research.

Various stakeholders at different levels have roles to play in building the evidence base on child marriage and utilizing it to advance policy and programmes to end child marriage and support married girls. At the country level, policy-makers can create fora for contextualizing global priorities to their local evidence needs and gaps, and determining which are most critical to advance national level change, and they can stimulate cross-sectoral efforts to fill these gaps and put the evidence generated to good use. Funders can invest in research that responds to local priorities, build local research capacity, and support more coordinated learning efforts. Programme managers can use the evidence generated by such efforts to inform their programmes, work with evaluators to assess the impact of their work, document lessons learned, and communicate with researchers regarding the questions they still need answered. Researchers can focus their efforts on the most important research gaps, conduct high quality research that meaningfully involves those most affected, and disseminate their findings in forms that are useful and accessible to other stakeholders. Only when these efforts are well connected will we see change on the scale needed to end child marriage and support married girls.

## Data Availability

Not applicable.

## Abbreviations

FGM: Female genital mutilation
HRP: UNDP-UNFPA-UNICEF-WHO-World Bank Special Programme of Research, Development and Research Training in Human Reproduction
VAW: Violence against women
WHO: World Health Organization
